# Real‐world data of chronic myelomonocytic leukemia: A chinese single‐center retrospective study

**DOI:** 10.1002/cam4.3774

**Published:** 2021-02-08

**Authors:** Liya Ma, Lingxu Jiang, Wenli Yang, Yingwan Luo, Chen Mei, Xinping Zhou, Gaixiang Xu, Weilai Xu, Li Ye, Yanlin Ren, Chenxi Lu, Peipei Lin, Jie Jin, Hongyan Tong

**Affiliations:** ^1^ Myelodysplastic Syndrome Center Department of Hematology The First Affiliated Hospital College of Medicine Zhejiang University Hangzhou China; ^2^ Dapartment of Radiotherapy Taizhou Central Hospital Taizhou Zhejiang China

**Keywords:** chemotherapy, chronic myelomonocytic leukemia, hypomethylating agents, leukemia‐free survival, overall response rate, overall survival

## Abstract

Chronic myelomonocytic leukemia (CMML) is a rare disease of elderly people characterized by the presence of sustained peripheral blood monocytosis, overlapping features of myeloproliferation, and myelodysplasia. We present a large retrospective study of 156 CMML patients in China. Mean age at diagnosis was 68 years old (range 23‐91). According to the CMML‐specific prognostic scoring system (CPSS), 10 patients (8.3%) were low risk, 27 patients (22.5%) were intermediate‐1 risk, 72 patients (60%) were intermediate‐2 risk, and 11 patients (9.2%) were high risk. A total of 90 patients (57.7%) received hypomethylating agents (HMAs) treatment, 19 patients (12.2%) received chemotherapy and 47 patients (30.1%) received the best supportive care. Seventeen patients (10.9%) underwent allogeneic hematopoietic stem cell transplantation (allo‐SCT) after HMAs treatment or chemotherapy. With a median follow‐up of 35.3 months, overall response rate (ORR) was 69.5% in the HMAs ± chemotherapy group, 79.5% in the HMAs monotherapy group, 60.0% in the HMAs + chemotherapy group, and 37.5% in the chemotherapy group. HMAs monotherapy group had prolonged OS compared with the chemotherapy group (23.57 months vs. 11.73 months; *p* = 0.035). Patients who achieved ORR had prolonged OS (25.83 months vs. 8.00 months; *p* < 0.001) and LFS (20.53 months vs. 6.80 months; *p* < 0.001) compared with those not achieved ORR in the HMA ± chemotherapy group. By univariate analysis, only higher hemoglobulin (≥80 g/L) and lower serum LDH levels (<300 U/L) predicted for better OS and LFS. By multivariate analysis, only Hb ≥ 80 g/L predicted for prolonged OS, Hb ≥ 80 g/L, and monocytes < 3 × 109/L predicted for prolonged LFS. In summary, our study highlights the benefit of HMAs therapy in CMML, but we still need to develop novel therapeutics to achieve better outcomes.

## Graphical abstract

Our retrospective study showed an advantage of HMAs therapy in CMML with an overall response rate of 65.9%‐79.5% compared with chemotherapy. The patients who respond to HMAs treatment have increased survival benefit compared to those who did not respond to HMAs treatment. HMAs monotherapy group had prolonged OS than the chemotherapy group.

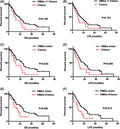

## 1 INTRODUCTION

Chronic myelomonocytic leukemia (CMML) is a clonal hematological malignancy characterized by peripheral blood monocytosis (≥1 × 10^9^/L, with monocytes ≥ 10% of the total white blood cells), and features of both myelodysplastic syndromes (MDS) and myeloproliferative neoplasms (MPN).^1^ CMML is a relatively rare disease with an approximated incidence of 1‐4 cases per million every year in western countries and a strong male preponderance. It is mainly diagnosed in older patients, with a median age of 71‐74 years at diagnosis.^2^, ^3^, ^4^, ^5^ The prognosis of CMML is poor with median overall survival ranging from 12 to 29 months and 14‐29% progression probability to acute myeloid leukemia (AML).^6^, ^7^, ^8^, ^9^

On the basis of WBC count, the French‐American‐British (FAB) classification divided CMML into two subtypes: myelodysplastic CMML (MD‐CMML) with WBC counts <13 × 10^9^/L and myeloproliferative CMML (MP‐CMML) with WBC counts ≥13 × 10^9^/L.^10^ Furthermore, 2016 WHO classification identifies three categories of CMML based on blasts of peripheral blood and bone marrow: CMML‐0 for patients with <2% blast in PB and <5% blast in BM; CMML‐1 for patients with 2‐4% blast in PB and/or 5‐9% blast in BM; and CMML‐2 for patients with 5‐19% in PB, 10‐19% in BM and/or presence of Auer rods.^1^

CMML is a highly heterogeneous disease with a distinctive biological diversity ranging from morphological changes, clinical manifestations, cytogenetic abnormalities, molecular biology and treatment responses.^11^ About 20‐30% of CMML patients have cytogenetic abnormalities, such as −Y, +8, del(20q), +21, −7, del(7q) and complex karyotype.^12^, ^13^, ^14^ Most CMML patients (>=90%) have gene mutations, frequent mutations including TET2 (~60%), SRSF2 (~50%), ASXL1 (~40%), and RAS (~30%).^15^

Until now, there is no agreement on the optimal therapy for CMML because of the high heterogeneity of the disease. Treatment modalities for CMML involved allogeneic hematopoietic stem cell transplantation (allo‐SCT), hypomethylating agents (HMAs), cytotoxic chemotherapy, and supportive care.^1^ Although allo‐SCT is the only available curative therapy, only a few patients are eligible due to advanced age and comorbidities.^16^, ^17^, ^18^

In the unfit patients for allo‐SCT, cytotoxic chemotherapy leads to low response rates and short response duration.^15^ Hydroxyurea is best suited for cytoreduction. HMAs such as decitabine and 5‐azacitidine have exhibited some effectiveness in postponing disease progression and were approved for the treatment of CMML.^19^, ^20^ Recently, several large retrospective studies demonstrated the overall response rates (ORR) of HMAs treatment ranged from 40% to 50% and complete remission rates (CR) were <20%.^21^

As there are limited data on CMML in China, even in Asia, we conducted a retrospective study to evaluate the variables that can influence the response rates. We analyzed the patients who received HMAs with or without chemotherapy as compared to the patients who received chemotherapy alone.

## 2 PATIENTS AND METHODS

### 2.1 Patients

We reviewed all the patients with a diagnosis of CMML in our hospital during a period from January 2003 to June 2019. One hundred and fifty‐six patients with peripheral monocytes >1 × 10^9^/L and bone marrow blast <19% were enrolled according to the 2016 WHO definition of CMML.^1^ Splenomegaly, hepatomegaly, and lymphadenectasis were defined as clinical or radiological enlargement. Bone marrow blasts contained promonocytes, myeloblasts, and agranular blasts. CMML‐specific prognostic scoring system (CPSS) was served to evaluate the prognosis of CMML patients.^22^ This study was approved by the Ethics Committee of the First Affiliated Hospital, College of Medicine, Zhejiang University, following principles of the Declaration of Helsinki.

### 2.2 Cytogenetic analysis

Cytogenetic analysis was performed in the Institute of Hematology in our hospital following standard protocols. In accordance with CMML‐specific cytogenetic risk classification, cytogenetic risk was categorized into three groups: low risk (normal karyotype or sole −Y), intermediate risk (all other abnormalities not in the high or low‐risk groups), and high risk (+8, abnormalities of chromosome 7, or complex karyotype).^13^

### 2.3 Gene sequencing analysis

Genomic DNA was extracted from bone marrow samples at diagnosis of CMML. A next‐generation sequencing (NGS) platform covering 185 genes was used to assess gene mutations in the patients diagnosed from 2015 to 2019. The gene panel included frequent genes in CMML: *TET2*, *ASXL1*, *IDH1*, *IDH2*, *DNMT3A*, *SRSF2*, *SF3B1*, *EZH2*, *ZRSR2*, *U2AF1*, *PTPN11*, *RUNX1*, *Tp53*, *CBL*, *JAK2*, *CSF3R*, *KRAS*, *NRAS*, *FLT3*, *MPL*, *KIT*, *CALR*, *NPM1*, *SETBP1*, *IKZF*, *CEBPA*, *and ETNK1*. A variant allele frequency (VAF) ≥ 2% was deemed as positive for analysis.

### 2.4 Treatment response

The Modified International Working Group (IWG 2006) response criteria^23^ were applied to evaluate treatment response. Categories of response contained complete remission (CR), partial remission (PR), marrow CR (mCR), hematologic improvement (HI), stable disease (SD), and treatment failure. The overall response rate (ORR) was the sum of CR, PR, and mCR or HI.

### 2.5 Follow‐up

The last follow‐up was performed on June 15th 2019. The median follow‐up period was 35.3 months (95%CI:14.44‐56.16). The overall survival (OS) was calculated as the period from the day of diagnosis to the day of death regardless of any cause or last contact. Leukemia‐free survival (LFS) was calculated from the day of diagnosis to the day of leukemia transformation or death, or last contact. The patients that underwent allo‐SCT were examined on the day of transplantation.

### 2.6 Statistical analysis

The SPSS 22.0 software (SPSS Inc.; Chicago, IL, USA) was used to conduct statistical analysis. Mann–Whitney U test was used to compare the baseline characteristics. Categorical variables were analyzed with the Chi‐square test. The analysis for predictors of HMAs response was carried out by means of logistic regression. P values less than 0.05 were regarded as statistically significant. OS and LFS curves were constructed by the Kaplan‐Meier method and compared by the log‐rank test. Factors associated with OS or LFS were analyzed first with univariate analysis, then followed by multivariate Cox proportional hazard regression.

## 3 RESULTS

### 3.1 Patient characteristics

A total of 156 patients diagnosed as CMML were enrolled in the study. Patient baseline characteristics, including age, gender, blood cell count, splenomegaly, hepatomegaly, lymphadenectasis, lactate dehydrogenase (LDH), bone marrow blasts, dysplasia lineages, cytogenetics, diagnostic classifications, and CPSS risk stratifications are shown in Table 1. Mean age at diagnosis was 68 years old (range 23‐91). One hundred (64.1%) patients were male. Seventy‐five patients (48.1%) had splenomegaly and 54 patients (34.6%) had lymphadenectasis, but only 6 patients (3.8%) had hepatomegaly. Median of LDH was 317 U/L. Median of bone marrow blasts was 8%. As for bone marrow dysplasia lineages, 28 patients (17.9%) involved with no lineage, 66 patients (42.3%) with one lineage, 45 patients (28.8%) with two lineages and 17 patients (10.9%) with all the three lineages. According to FAB classification, 54 patients (35.1%) were considered to have dysplastic subtype and 100 patients (64.9%) were considered to have proliferative subtype.^10^ Overall, 42 patients (26.9%) belonged to CMML‐0, 46 patients (29.5%) to CMML‐1 and 68 patients (42.6%) to CMML‐2 according to 2016 WHO classification.^1^

**TABLE 1:** Clinical and laboratory characteristics of 156 patients with chronic myelomonocytic leukemia (CMML)

Variables	Baseline distribution in cohort *N* = 156
Age (years), median (range)	68 (23‐91)
Male gender, *N* (%)	100 (64.1)
Splenomegaly, *N* (%)	75 (48.1)
Hepatomegaly, *N* (%)	6 (3.8)
Lymphadenectasis, *N* (%)	54 (34.6)
Blood counts, median (range)	
Hb (g/dL)	8.6 (4.5‐15.3)
Platelets (×10^9^/L)	66 (3‐1344)
WBC (×10^9^/L)	17.7 (3.6‐200.0)
ANC (×10^9^/L)	8.3 (0.9‐130.0)
Monocytes (×10^9^/L)	3.8 (1.0‐51.3)
Bone marrow blasts (%), median (range)	8.0 (0.0‐19.5)
Lineages of marrow dysplasia, *N* (%)	
0	28 (17.9)
1	66 (42.3)
2	45 (28.8)
3	17 (10.9)
LDH (U/L), median (range)	317 (114‐2000)
FAB classification (*N* = 154), *N* (%)	
MD‐CMML	54 (35.1)
MP‐CMML	100 (64.9)
2016 WHO classification, *N* (%)	
CMML‐0	42 (26.9)
CMML‐1	46 (29.5)
CMML‐2	68 (43.6)
Cytogenetic risk (*N* = 123), *N* (%)	
Low	80 (65.0)
Intermediate	25 (20.3)
High	18 (14.6)
CPSS (*N* = 120)	
Low	10 (8.3)
Intermediate‐1	27 (22.5)
Intermediate‐2	72 (60.0)
High	11 (9.2)
First line of treatment, *N* (%)	
Best supportive care	47 (30.1)
HMAs	90 (57.7)
Chemotherapy	19 (12.2)
Reception of HSCT, *N* (%)	17 (10.9)
Outcome, *N* (%)	
Leukemic transformation	27 (17.3)
Death	86 (55.1)

Abbreviations: ANC: absolute neutrophil count; CMML: chronic myelomonocytic leukemia; CPSS: CMML‐specific prognostic scoring system; FAB: French‐American‐British; Hb: Hemoglobin; HMAs: hypomethylating agents; HSCT: hematopoietic stem cell transplantation; LDH: lactate dehydrogenase;WBC: white blood cells; WHO: World Health Organization.

Karyotype was available in 123 patients. Among them, 43 patients (35%) carried with abnormal karyotype: 8 patients (18.60%) with complex karyotype, 4 patients (9.30%) with +8, 2 patients (4.65%) with del(20q), 2 patients (4.65%) with −5 or del(5q), 2 patients (4.65%) with −7 or del(7q), 2 patients (4.65%) with +21, 2 patients (4.65%) with −18, 1 patient (2.32%) with −12, and 1 patient (2.32%) with −Y. In accordance with the cytogenetic risk stratification, 80 patients (65%) categorized to the low‐risk group, 25 patients (20.3%) to the intermediate risk group, and 18 patients (14.6%) to the high‐risk group (Table 1).

Thirteen patients had gene sequencing results and all of them were identified gene mutations. The frequency of gene mutations were *ASXL1* (*n* = 4, 30.7%), *DNMT3A* (*n* = 4, 30.7%), *U2AF1* (*n* = 3, 23.1%), *TP53* (*n* = 3, 23.1%), *PTPN11* (*n* = 3, 23.1%), *NPM1* (*n* = 3, 23.1%), *TET2* (*n* = 2, 15.4%), *RUNX1* (*n* = 2, 15.4%), *NRAS* (*n* = 2, 15.4%), *KMT2D* (*n* = 2, 15.4%), *KRAS* (*n* = 2, 15.4%), *CSF3R* (*n* = 2, 15.4%), *SETBP1* (*n* = 1, 7.7%), *PHF6* (*n* = 1, 7.7%), *BCOR* (*n* = 1, 7.7%) and *CBL* (*n* = 1, 7.7%).

Following the CMML‐specific prognostic scoring system (CPSS), 10 patients (8.3%) were low‐risk, 27 patients (22.5%) were intermediate‐1 risk, 72 patients (60%) were intermediate‐2 risk, and 11 patients (9.2%) were high‐risk.

### 3.2 Treatment modalities

Among the patients who were treated with HMAs, 81 patients treated with decitabine and 9 patients treated with azacitidine (Table 1). The median number of cycles of HMAs therapy was four cycles (range 1–17). The median number of cycles of chemotherapy was two cycles (range 1–4). Seventeen patients (10.9%) underwent allo‐SCT after HMA treatment or chemotherapy. Six patients (35.3%) died after transplantation: three patients died of SCT related complications in the early stage, three patients transformed to acute myeloid leukemia and died of salvage therapy failure.

### 3.3 Treatment response

We compared the baseline data between the HMAs ± chemotherapy group (including HMAs monotherapy and HMAs combined chemotherapy) and chemotherapy group. As shown in Table 2, HMAs ± chemotherapy group had lower white blood cells (16.7 × 10^9^/L vs. 33.8 × 10^9^/L, *p* < 0.001), lower neutrophils (7.7 × 10^9^/L vs. 16.0 × 10^9^/L, *p* = 0.005), lower monocytes (3.5 × 10^9^/L vs. 9.3 × 10^9^/L, *p* = 0.005), lower percent of bone marrow blasts (8.8% vs. 14.0%, *p* = 0.020), lower LDH (306 U/L vs. 408 U/L, *p* = 0.034) and lower percent of MP‐CMML patients (59.8% vs. 87.5%, *p* = 0.034) in comparison to chemotherapy group. However, on a multivariable analysis including adjusted age (≥70 vs. <70), WBC (≥10 × 10^9^/L vs. <10 × 10^9^/L), neutrophils (≥1 × 10^9^/L vs. <1 × 10^9^/L), monocytes (≥3 × 10^9^/L vs. <3 × 10^9^/L), hemoglobulin (≥80 g/dL vs. <80 g/dL), platelets (≥50 × 10^9^/L vs. <50 × 10^9^/L), bone marrow blasts (≥5% vs. <5%) and LDH (≥300 U/L vs. <300 U/L), HMAs+/‐chemotherapy remained to be associated with higher ORR compared with chemotherapy (OR: 3.333, 95% CI 1.09l6–10.141; *p* = 0.034).

**TABLE 2:** Characteristics of the patients treated with hypomethylating agents and chemotherapy

Variables	HMAs+/chemo, *N* (%), *N* = 82	Chemotherapy, N (%) *N* = 16	*p*
Age (years), median (range)	64.5 (26‐90)	71 (45‐86)	0.260
Male gender, *N* (%)	59 (72.0)	9 (56.3)	0.242
Splenomegaly, *N* (%)	37 (45.1)	11 (68.8)	0.084
Hepatomegaly, *N* (%)	4 (4.9)	1 (6.3)	1.000
Lymphadenectasis, *N* (%)	30 (36.6)	5 (31.3)	0.684
Blood counts, median (range)			
Hb (g/L)	85 (45‐151)	77 (59‐127)	0.563
Platelets (×10^9^/L)	66 (4‐1344)	40 (3‐334)	0.104
WBC (×10^9^/L)	16.7 (3.6‐132.9)	33.8 (9.2‐200.0)	<0.001
ANC (×10^9^/L)	7.7 (0.9‐120.4)	16.0 (5.4‐130.0)	0.005
Monocytes (×10^9^/L)	3.5 (1.0‐51.3)	9.3 (1.0‐50.0)	0.005
Bone marrow blasts (%), median (range)	8.8 (1.0‐19.5)	14.0 (4.0‐19.50)	0.020
Lineages of marrow dysplasia, N (%)			0.635
0	16 (19.5)	1 (6.3)	
1	33 (40.2)	8 (50.0)	
2	24 (29.3)	5 (31.3)	
3	9 (11.0)	2 (12.5)	
LDH (U/L), median (range)	306 (114‐2000)	408 (346‐882)	0.034
FAB classification (*N* = 98), *N* (%)			0.034
MD‐CMML	33 (40.2)	2 (12.5)	
MP‐CMML	49 (59.8)	14 (87.5)	
2016 WHO classification, *N* (%)			0.058
CMML−0	17 (20.7)	1 (6.3)	
CMML−1	25 (30.5)	2 (12.5)	
CMML−2	40 (48.8)	13 (81.3)	
Cytogenetic risk (*N* = 83), *N* (%)			0.392
Low	47 (65.3)	5 (45.5)	
Intermediate	15 (20.8)	3 (27.3)	
High	10 (13.9)	3 (27.3)	

First, we compared the treatment response between HMAs ± chemotherapy group and chemotherapy group (Table 3). According to the IWG 2006 response criteria, HMAs ± chemotherapy response was accessed in 82 patients including: 10 CR (12.2%), 43 mCR or HI (52.4%), 1 PR (1.2%), 7 SD (4.5%), 15 PD (9.6%), and 6 failures (3.8%). Chemotherapy response was available in 16 patients including: 0 CR, 6 mCR (37.5%), 0 PR, 2 SD (12.5%), 5 PD (31.3%) and 3 failures (18.8%). Patients treated with HMAs ± chemotherapy achieved significantly higher ORR than chemotherapy group (65.9% vs. 37.5%, *p* = 0.033).

**TABLE 3:** Treatment response using hypomethylating agents versus chemotherapy

Response assessment	HMAs ± chemo, *N* (%) *N* = 82	Chemotherapy, *N* (%) *N* = 16	*p*
CR	10 (12.2)	0 (0)	0.360
PR	1 (1.2)	0 (0)	1.000
mCR/HI	43 (52.4)	6 (37.5)	0.413
SD	7 (4.5)	2 (12.5)	0.638
DP	15 (9.6)	5 (31.3)	0.308
Failure	6 (3.8)	3 (18.8)	0.161
ORR (CR + PR + mCR/HI)	54 (65.9)	6 (37.5)	0.033

Abbreviations: CR: complete remission, PR: partial remission, mCR/HI: marrow complete remission/hematologic improvement, SD: stable disease, DP: disease progression; Failure: treatment failure, ORR: overall response rate (CR+PR+mCR/HI); HMAs+/‐chemo: HMAs monotherapy and HMAs combined chemotherapy.

Second, we compared treatment response between the HMAs monotherapy group and chemotherapy group (Table 4). ORR was 79.5%, including 5 CR (8.1%), 36 mCR/HI (58.1%), and 1 PR (1.6%) in HMAs monotherapy group. In contrast, ORR was 37.5% (6 patients achieved mCR/HI and no patients achieved CR or PR) in the chemotherapy group. There was a noteworthy difference in ORR between the two groups (*p* = 0.027).

**TABLE 4:** Treatment response using HMAs monotherapy versus chemotherapy

Response assessment	HMAs mono, *N* (%) *N* = 62	Chemotherapy, *N* (%) *N* = 16	*p*
CR	5 (8.1)	0 (0)	0.577
PR	1 (1.6)	0 (0)	1.000
mCR/HI	36 (58.1)	6 (37.5)	0.141
SD	6 (9.7)	2 (12.5)	0.664
DP	13 (21.0)	5 (31.3)	0.506
Failure	1 (1.6)	3 (18.8)	0.026
ORR (CR + PR + mCR/HI)	42 (79.5)	6 (37.5)	0.027

Finally, we compared treatment response between the HMAs monotherapy group and HMAs+chemotherapy group (Table 5). ORR was 60.0% including 5 with CR (25%), 7 mCR/HI (35%), and 0 PR in the HMAs + chemotherapy group. There was no statistical difference in ORR between the two groups (79.5% vs. 60.0%, *p* = 0.526).

**TABLE 5:** Treatment response using HMAs monotherapy versus HMAs + chemotherapy

Response assessment	HMAs mono, *N* (%) *N* = 62	HMAs + chemo, *N* (%) *N* = 20	*p*
CR	5 (8.1)	5 (25.0)	0.058
PR	1 (1.6)	0 (0)	1.000
mCR/HI	36 (58.1)	7 (35.0)	0.073
SD	6 (9.7)	1 (5.0)	1.000
DP	13 (21.0)	2 (10.0)	0.339
Failure	1 (1.6)	5 (25.0)	0.003
ORR (CR + PR + mCR/HI)	42 (79.5)	12 (60.0)	0.526

### 3.4 Survival

**FIGURE 1:**
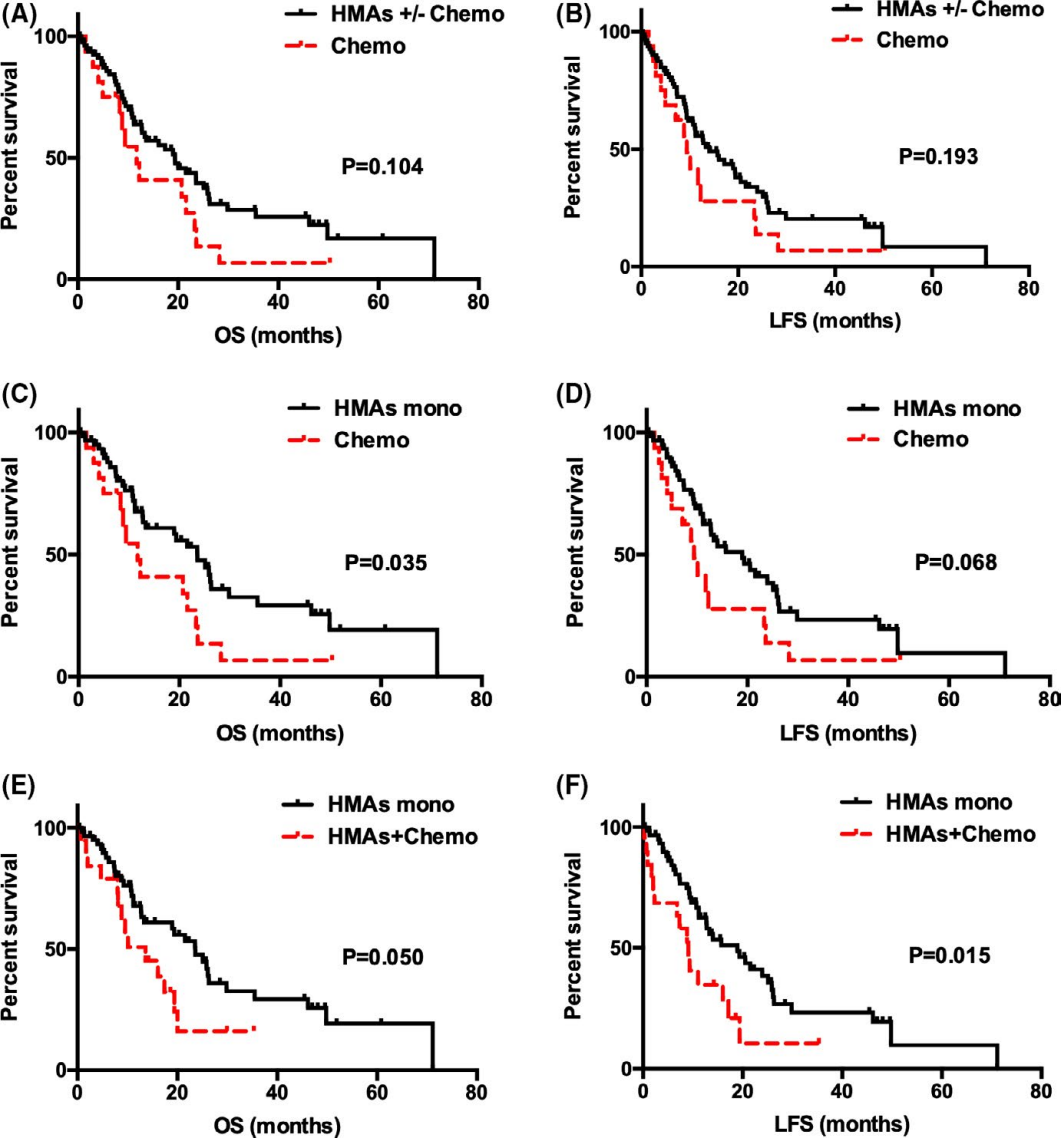
Overall and leukemia free survival among different treatment groups. (A) Overall survival in two groups: HMAs ± chemotherapy versus chemotherapy (19.37 vs 11.73 months; *p* = 0.104); (B) Leukemia free survival in two groups: HMAs ± chemotherapy versus chemotherapy (14.00 vs 9.40 months; *p* = 0.193); (C) Overall survival in two groups: HMAs monotherapy versus chemotherapy (23.57 vs 11.73 months; *p* = 0.035); (D) Leukemia free survival in two groups: HMAs monotherapy versus chemotherapy (18.97 vs 9.40 months; *p* = 0.068); (E) Overall survival in two groups: HMAs monotherapy versus HMAs + chemotherapy (23.57 vs 13.60 months; *p* = 0.050); (F) Leukemia free survival in two groups: HMAs monotherapy versus HMAs + chemotherapy (18.97 vs 9.10 months; *p* = 0.015).

The median follow‐up period was 35.3 months (95% CI: 14.44‐56.16) and nine cases were lost to follow‐up. Median OS was 23.3 months (95% CI: 20.23–‐26.37) and median LFS was 19.4 months (95% CI: 13.33‐25.47). Twenty‐seven patients (16.7%) developed a transformation into acute myelocytic leukemia, verified by bone marrow aspiration and flow cytometry. OS was not significantly different between the two groups (19.37 months in HMAs ± chemotherapy group vs. 11.73 months in the chemotherapy group, *p* = 0.104) (Figure 1A), but the HMAs ± chemotherapy group tended to have prolonged OS. There was no apparent difference between the two groups in LFS (14.0 months in HMAs ± chemotherapy group vs. 9.40 months in the chemotherapy group, *p* = 0.193) (Figure 1B).

However, the HMAs monotherapy group had prolonged OS than the chemotherapy group (23.57 months vs. 11.73 months; *p* = 0.035) (Figure 1C), though LFS showed no difference between the two groups (18.97 months vs. 9.40 months; *p* = 0.068) (Figure 1D). HMAs monotherapy group had no advantage on OS compared with the HMAs + chemotherapy group (23.57 months vs. 13.60 months; *p* = 0.050) (Figure 1E), but had superiority on LFS (18.97 months vs. 9.10 months; *p* = 0.015) (Figure 1F).

**FIGURE 2:**
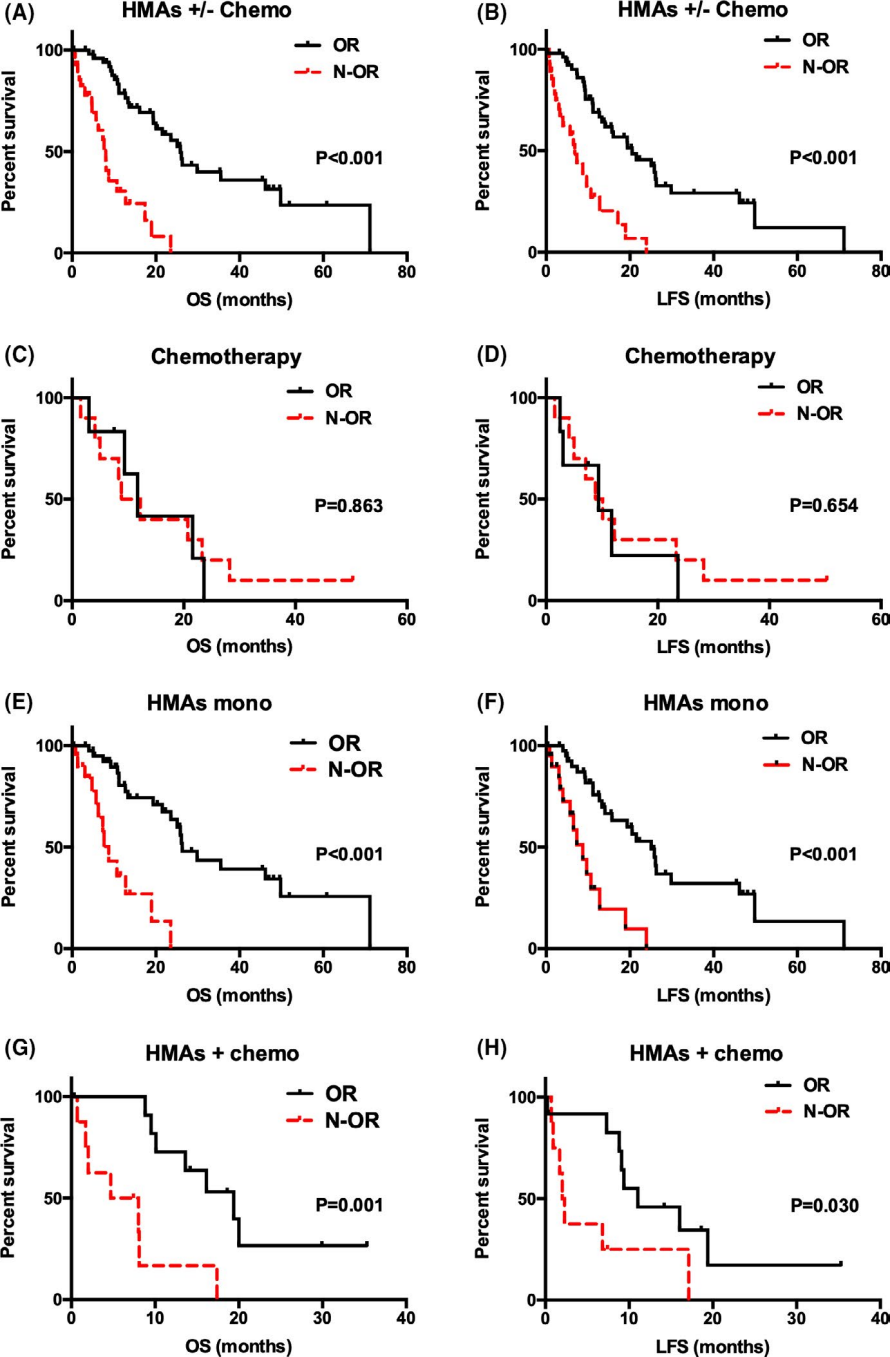
Comparison of OS and LFS between the patients achieved ORR and the patients not achieved ORR. (A) Median OS of patients treated with HMAs ± chemotherapy achieved ORR versus Non‐ORR: 25.83 vs 8.00 months; *p* < 0.001; (B) Median LFS of patients treated with HMAs ± chemotherapy achieved ORR versus Non‐ORR: 20.53 vs 6.80 months; *p* < 0.001; (C) Median OS of patients treated with chemotherapy achieved ORR versus Non‐ORR: 11.73 vs 10.52 months; *p* = 0.863; (D) Median LFS of patients treated with chemotherapy achieved ORR versus Non‐ORR: 9.40 vs 9.47 months; *p* = 0.654; (E) Median OS of patients treated with HMAs monotherapy achieved ORR versus Non‐ORR: 26.27 vs 8.73 months; *p* < 0.001; (F) Median LFS of patients treated with HMAs monotherapy achieved ORR versus Non‐ORR: 25.1 vs 8.73 months; *p* < 0.001; (G) Median OS of patients treated with HMAs + monotherapy achieved ORR versus Non‐ORR: 19.40 vs 6.35 months; *p* = 0.001; (H) Median LFS of patients treated with HMAs + monotherapy achieved ORR versus Non‐ORR: 11.03 vs 2.14 months; *p* = 0.030.

Patients who achieved ORR had prolonged OS (25.83 months vs. 8.00 months; *p* < 0.001) (Figure 2A) and LFS (20.53 months vs. 6.80 months; *p* < 0.001) than those not achieved ORR in HMAs ± chemotherapy group (Figure 2B). Similarly, patients who achieved ORR had prolonged OS (26.27 months vs. 8.73 months; *p* < 0.001) (Figure 2E) and LFS (25.1 months vs. 8.73 months; *p* < 0.001) (Figure 2F) than those not achieved ORR in HMAs monotherapy group. Patients who achieved ORR also had prolonged OS (19.40 months vs. 6.35 months; *p* = 0.001) (Figure 2G) and LFS (11.03 months vs. 2.14 months; *p* = 0.030) (Figure 2H) than those not achieved ORR in the HMAs + chemotherapy group. Conversely, the OS of patients in the chemotherapy group had no relationship with whether they achieved ORR or not (11.73 months vs. 10.52 months; *p* = 0.863) (Figure 2C), neither the LFS of them (9.40 months vs. 9.47 months; *p* = 0.654) (Figure 2D).

### 3.5 Subgroup analysis

As shown in Table 6, subgroup analyses based on age, monocytes, platelets indicated age < 70 years, monocytes < 3 × 10^9^/L, PLT ≥ 50 × 10^9^/L had no impact on OS or LFS. Nonetheless, A subgroup analysis based on hemoglobin demonstrated that Hb ≥ 80 g/L had a remarkable impact on OS (Hb ≥ 80 g/L: 28.23 months vs. Hb < 80 g/L: 12.70 months; *p* < 0.001) or LFS (Hb ≥ 80 g/L: 25.1 months vs. Hb < 80 g/L: 9.67 months; *p* < 0.001). Furthermore, a subgroup analysis based on LDH manifested a relation of LDH ≥ 300 U/L with poor OS (LDH < 300 U/L: 29.83 months vs. LDH ≥ 300 U/L: 16.13 months; *p* < 0.001) as well as poor LFS (LDH < 300 U/L: 25.20 months vs. LDH ≥ 300 U/L: 11.20 months; *p* < 0.001). In contrast, splenomegaly, hepatomegaly, lymphadenectasis, FAB subtypes, WHO subtypes, cytogenetic risk stratification, and CPSS stratification had no influence in OS or LFS.

**TABLE 6:** Subgroup analysis of OS and LFS

Variables	OS (months)	*p*	LFS (months)	*p*
Age (years)		0.714		0.166
<70	23.50		15.70	
≥70	21.57		21.53	
Monocytes		0.279		0.115
<3 × 10^9^/L	23.57		20.53	
≥3 × 10^9^/L	21.53		14	
PLT		0.442		0.557
≥50 × 10^9^/L	23.3		19.4	
<50 × 10^9^/L	20		16.03	
Hb		<0.001		<0.001
≥80 g/L	28.23		25.1	
<80 g/L	12.70		9.67	
LDH		<0.001		<0.001
<300 U/L	29.83		25.20	
≥300 U/L	16.13		11.20	
Splenomegaly		0.298		0.438
Yes	20		18.97	
No	23.63		19.70	
Hepatomegaly		0.234		0.356
Yes	11.42		10.13	
No	23.30		19.40	
Lymphadenectasis		0.408		0.487
Yes	21.53		13.33	
No	23.57		19.40	
FAB classification		0.092		0.156
MD‐CMML	24.43		23.63	
MP‐CMML	19.40		15.47	
2016 WHO classification		0.181		0.089
CMML‐0	19.70		19.70	
CMML‐1	24.93		24.43	
CMML‐2	20.00		12.23	
Cytogenetic risk		0.693		0.756
Low	23.63		21.53	
Intermediate	23.57		20.53	
High	20.70		10.13	
CPSS stratification		0.514		0.360
Low	not reached		not reached	
Intermediate‐1	23.30		23.30	
Intermediate‐2	23.63		18.97	
High	20.70		9.67	
Transplant		0.144		0.870
Yes	Not reached		17.13	
No	21.57		19.40	

Median OS after transplant was not reached for the patients who underwent allo‐SCT. But there was no significant difference in OS (transplant: not reached vs. non‐transplant: 21.57 months; *p* = 0.144) or LFS (transplant: 17.13 months vs. non‐transplant: 19.40 months; *p* = 0.870) between the transplant patients and non‐transplant patients.

On a univariate analysis that included adjusted age, monocytes, hemoglobulin, platelets, LDH, splenomegaly, hepatomegaly, lymphadenectasis, FAB subtypes, CPSS stratification, and transplant, only Hb < 80 remained to be related with shorter OS (HR: 2.194, 95% CI 1.253‐3.839; *p* = 0.006). On a multivariable analysis that included these aforementioned factors, Hb < 80 (HR: 2.864, 95% CI 1.707‐4.805; *p* < 0.001) and monocytes ≥ 3 × 10^9^/L (HR: 1.796, 95% CI 1.028‐3.139; *p* = 0.040) remained to be associated with shorter LFS.

## 4 DISCUSSION

In this study, we present a large retrospective study of 156 patients with CMML in China. Our results suggest that HMAs therapy with or without chemotherapy has a superior outcome. Median age at diagnosis in our cohort was younger than the published literature.^2^, ^3^, ^4^, ^5^ Almost half of the patients had splenomegaly and nearly one‐third of the patients had lymphadenectasis. The LDH (317 U/L) was much higher than normal. The majority of the patients had one or two dysplasia lineages in bone marrow. From the proportion of patients, MP‐CMML patients accounted for the majority.

Similar to previous studies,^12^, ^13^, ^14^, ^24^ two‐thirds of our patients had normal karyotype, while one‐third of the patients had chromosome abnormalities in our study. The most frequent cytogenetic abnormality was complex karyotype, so quite a few of patients were categorized into the high‐risk group.

From the treatment history, most patients diagnosed before 2010 received chemotherapy. After that, decitabine was widely used to treat CMML alone or combined with chemotherapy in our hospital. Since 2017, azacitidine was applied to the treatment of CMML due to its entry into medical insurance in China. As a result, the majority of patients received decitabine treatment and only a few patients received azacytidine treatment in our study.

In recent years, several trials have accessed the efficacy of HMAs treatment in CMML patients. ORR ranged from 25% to 70% and CR ranged from 10% to 58% in these studies.^21^ Median OS ranged from 12 to 37 months. These studies indicated a definite benefit of HMAs in CMML.^25^ In our study, the ORR was 79.5% and CR was 8.1% in the HMAs monotherapy group. The ORR was 65.9% and CR was 12.2% in the HMAs ± chemotherapy group. The patients treated with HMAs ± chemotherapy and HMA monotherapy achieved significantly higher ORR than the chemotherapy group. Taken together, HMA‐based treatment demonstrated an obvious advantage in comparison to chemotherapy in our study.

Our study showed that the HMAs monotherapy group had prolonged OS than the chemotherapy group. Conversely, the OS and LFS of patients in the chemotherapy group had no relationship with whether they achieved ORR or not probably because they all have poor prognosis.

By univariate analysis, only higher hemoglobulin (≥80 g/L) and lower serum LDH levels (<300 U/L) predicted for better OS and LFS. By multivariate analysis, only Hb ≥ 80 g/L retained prognostic significance on OS, Hb ≥ 80 g/L, and monocytes < 3 × 10^9^/L retained prognostic significance on LFS. These results were like other previously published studies.^25^, ^26^

Despite allo‐SCT is the solely potentially curative therapy, only 17 patients (10.9%) went through allo‐SCT after HMA treatment or chemotherapy in our cohort. The patients received allo‐SCT tended to have longer OS and LFS than non‐transplant patients. However, there was no statistical difference between the transplant patients and the non‐transplant patients in OS due to the high mortality rate of allo‐SCT (35.3% of patients died after transplantation).

Our study has some limitations. First, our data represented a single‐center retrospective study. Moreover, because we incorporated into the study with CMML patients diagnosed from 2003 to 2019, only 13 patients had gene sequencing data (NGS available since 2015). Therefore, we could not estimate the prognosis of all our patients with CPSS‐mol stratification which integrates conventional indicators and gene mutations, ameliorates the risk stratification of CMML.^7^ Finally, we could not assess response with the 2015 International Consortium Response Criteria for Myelodysplastic/Myeloproliferative neoplasm^27^ which contains “clinical benefit” because detailed information in accordance with the criteria, were unavailable in most patients.

In conclusion, our retrospective study showed an advantage of HMAs therapy in CMML with ORR of 65.9%‐79.5% compared with chemotherapy. The patients who respond to HMAs treatment had longer survival compared to those who did not respond to HMAs treatment. HMAs monotherapy group had prolonged OS than the chemotherapy group. Although HMAs treatment achieved high response but could not significantly modify the disease process. There is still an unslaked need for other therapy that could improve response rates, alter the disease process and prolong survival.

## 5 AUTHOR CONTRIBUTION STATEMENT

Liya Ma designed the study and wrote the paper; Lingxu Jiang, Wenli Yang, and Yingwan Luo collected the data; Chen Mei, Xinping Zhou analyzed the data; Gaixiang Xu, Weilai Xu, and Li Ye did the statistical analysis; Yanlin Ren, Chenxi Lu, and Peipei Lin completed the follow‐up; Jie Jin edited the paper; Hongyan Tong reviewed and revised the paper.

## CONFLICT OF INTEREST

The authors state that they have no conflict of interest.

## Data Availability

The authors affirm that the data supporting the findings of this study are available within the article.
